# International Heart Valve Bank Survey: A Review of Processing Practices and Activity Outcomes

**DOI:** 10.1155/2013/163150

**Published:** 2013-09-15

**Authors:** Wee Ling Heng, Helmi Albrecht, Paul Chiappini, Yeong Phang Lim, Linda Manning

**Affiliations:** ^1^National Cardiovascular Homograft Bank, Department of Cardiothoracic Surgery, National Heart Centre Singapore, Singapore 168752; ^2^Sydney Heart Valve Bank, St. Vincent's Hospital, 390 Victoria Street, Darlinghurst, NSW 2010, Australia; ^3^Cell and Tissue Therapies WA, Royal Perth Hospital, 197 Wellington Street, Perth, WA 6000, Australia

## Abstract

A survey of 24 international heart valve banks was conducted to acquire information on heart valve processing techniques used and outcomes achieved. The objective was to provide an overview of heart valve banking activities for tissue bankers, tissue banking associations, and regulatory bodies worldwide. Despite similarities found for basic manufacturing processes, distinct differences in procedural details were also identified. The similarities included (1) use of sterile culture media for procedures, (2) antibiotic decontamination, (3) use of dimethyl sulfoxide (DMSO) as a cryoprotectant, (4) controlled rate freezing for cryopreservation, and (5) storage at ultralow temperatures of below −135°C. Differences in procedures included (1) type of sterile media used, (2) antibiotics combination, (3) temperature and duration used for bioburden reduction, (4) concentration of DMSO used for cryopreservation, and (5) storage duration for released allografts. For most banks, the primary reasons why allografts failed to meet release criteria were positive microbiological culture and abnormal morphology. On average, 85% of allografts meeting release criteria were implanted, with valve size and type being the main reasons why released allografts were not used clinically. The wide variation in percentage of allografts meeting release requirements, despite undergoing validated manufacturing procedures, justifies the need for regular review of important outcomes as cited in this paper, in order to encourage comparison and improvements in the HVBs' processes.

## 1. Introduction

Since the first heart valve bank (HVB) started in New Zealand in 1962, the recovery, processing, and storage techniques have been constantly evolving to improve the quality and safety of cardiovascular allografts for clinical implantation. During the initial era of allograft usage, fresh aseptically recovered allografts were implanted within hours or days of recovery. This was followed by aggressive decontamination methods, such as gamma-irradiation and chemical sterilisation using formaldehyde, glutaraldehyde, beta-propiolactone, and ethylene oxide. Coupled with harsh preservation techniques of freeze-drying and flash-freezing in liquid nitrogen, these “sterilised” tissues rapidly failed *in situ* due to high incidence of leaflet degeneration, cusp rupture, and/or loss of durability and hemodynamic function. For these reasons, allograft use was discontinued until newer preservation methods were developed. Eventually, “sterilised” tissues were replaced by aseptically recovered ones treated with antibiotics and stored in culture media at 4°C for up to 6 weeks. These milder techniques improved valve durability and, ultimately, patient outcome. Today, the majority of HVBs worldwide use aseptic retrieval of donor heart valves followed by low-dose antibiotic decontamination, cryopreservation, and storage at ultralow temperature until the valves are required for implantation [1–3].

Regulatory bodies throughout the world promote global harmonisation of manufacturing procedures as a mean to standardise product quality and safety and to simplify the exchange of “like” products between jurisdictions. Although standardisation is a rational approach for many processes, genuine differences and restrictions in manufacturing circumstances, such as differences in endemic microbial contaminants and patented processes, may limit the extent to which standardisation can be achieved. In addition, as changes to critical processes require validation, which can be both costly and time consuming, any proposed change must be justified in terms of risks and cost benefits for the HVB and the recipient. As a result, even though tissue banking associations and regulatory bodies governing HVBs worldwide promote similar quality and safety standards for allografts, differences in processing procedures used to achieve these outcomes continue to exist. Essentially, all HVBs follow standards developed by their regional tissue banking associations, which are designed to meet regulatory requirements of their jurisdiction. For instance, in North America, most HVBs follow the standards of the American Association of Tissue Banks (AATB). The European Association of Tissue Banks (EATB), British Association for Tissue Banking (BATB), Associación Español de Bancos de Tejidos (AEBT), and the Spanish Association of Tissue Banks (SATB) have published tissue banking standards for European banks [4]. In Australia, the Australasian Tissue and Biotherapeutics Forum (ATBF) has developed standards that align with the Therapeutic Goods Administration's (TGA) new Biologicals Regulatory Framework. In Asia, the Asia-Pacific Association of Surgical Tissue Banking (APASTB) was formed to encourage tissue-focused research and to promote scientific and social interaction among its members. In all cases, the standards developed by these tissue-banking associations stipulate donor suitability criteria, aseptic processing procedures, and controlled storage requirements of transplantable human tissues, with the common objective of assuring that recipients receive disease- and contaminant-free allografts that fulfil optimum clinical performance [2].

In February 2011, a survey of 24 HVBs in North America, Europe, Australasia, and South Africa was initiated by coauthor Linda Manning. The survey was designed to collate data on heart valve processing procedures and outcomes to inform HVBs and their regulatory bodies on the similarities and differences in heart valve manufacturing activities worldwide. This information was subsequently compiled and presented at the ATBF's 12th Annual Scientific and Business meeting held in Melbourne, Australia, in May 2011 and the AATB's 35th Annual Meeting held in Arizona, USA, in September 2011. This paper reports the comprehensive data collected from individual HVBs as part of this survey.

## 2. Materials and Methods

The original survey was distributed by electronic mail to 24 HVBs in North America, Europe, Australia, New Zealand, Asia, and South Africa. They were asked to provide information to the following questions, which addressed two aspects.

1. Allograft processing and storage procedures:
  - what processing media does your bank use?
  - what antibiotic regimen does your bank use?
  - what cryopreservation method does your bank use?
  - did your bank conduct a formal validation on the media, antibiotics, and antibiotic incubation protocols that you use?
  - what storage conditions and duration does your bank use for allografts meeting release criteria?
2. Processing outcome of HVBs:
  - how many donors and allografts does your bank process annually?
  - what proportion of allografts meet release criteria?
  - what are the most common reasons allografts fail to meet release criteria?
  - what proportion of allografts released for clinical use is implanted?
  - what are the primary reasons why released allografts are not implanted?

Data was collated from February to April 2011. In this report, the results were summarised and presented as returned, apart for editing for consistency of terminology. The individual HVBs had been deidentified. The Royal Perth Hospital's Cell and Tissue Therapies WA's Management Committee was consulted prior to implementation of study, and ethics approval was advised to be unnecessary, as the survey was a mere collation of information on the individual HVBs' practices and outcomes, which did not involve the identification of patients.

## 3. Results

### 3.1. Tissue Processing and Storage Procedures

24 HVBs participated in this aspect of study. As presented in Tables 1, 2, and 3, both similarities and differences in heart valve processing and storage procedures were identified in the survey. Isotonic solutions or cell culture media were used for valve retrieval, antibiotic decontamination, and cryopreservation. Eleven HVBs (46%) used Medium 199 (M199), and five (21%) used Roswell Park Memorial Institute Media 1640 (RPMI-1640), while the remainder used Dulbecco's Modified Eagle's Medium (DMEM), sodium chloride (normal saline), Hanks Balanced Salt Solution (HBSS), or Ringer's lactate (Table 5). Twelve of the 18 HVBs (66%) reported using 9%–11% DMSO as cryoprotectant, with the rest using varying concentrations of DMSO (7.5%–20%) with or without foetal bovine serum or human albumin. In all cases, the media/solutions were purchased as sterile reagents from approved suppliers.

Antibiotic decontamination regimens were found to be exceptionally diverse, both in the antibiotic combinations used (Tables 1–3) and their concentrations (Table 4). The most commonly used antibiotics were vancomycin (15 banks) and gentamicin (10 banks). Seven HVBs also included antifungal drugs in their antibiotic cocktail-five used amphotericin B, one used fluconazole, and another used fungoral.

Most of the European HVBs and half of the North American HVBs surveyed incubated allografts at 4°C for 24 hours. In contrast, five of the seven HVBs surveyed from Australasia and South Africa decontaminated their valves at physiological temperature of 37°C for ≤12 hours. Overall, 16 HVBs (67%) conducted bioburden reduction at cold temperatures (1°C–10°C), with seven HVBs (29%) incubating at 33°C–38°C. Duration of bioburden reduction step varied from 6 to 48 hours, with a majority of the HVBs incubating for 18–26 hours at cold temperatures (12 of 16 banks = 75%). Those using physiological temperatures (33°C–38°C) incubated for either 18–26 hours (3 of 7 banks = 43%) or 6–12 hours (4 of 7 banks = 57%). Two HVBs conducted bioburden reduction at ambient temperature for 24 hours. One HVB decontaminated allografts at either 37°C for 6–8 hours or 4°C for 18–24 hours (Table 6).

92% of HVBs (22 of 24 banks) used controlled rate freezing to cryopreserve cardiovascular tissues, with the remaining two HVBs using manual freezing in liquid nitrogen vapour phase. All HVBs stored processed allografts at ultralow temperatures (<−135°C) or in liquid nitrogen vapour phase. Storage duration ranged from 2 to 15 years, with the majority of HVBs (16 of 23 banks = 70%) storing released homografts for five years.

The procedures used for processing, bioburden reduction, cryopreservation, and storage had been validated by all responding HVBs.

### 3.2. HVBs' Processing Activities and Outcomes

23 HVBs participated in this aspect of study. A summary of the heart valve processing activities and outcomes is presented in Tables 7, 8, and 9. The annual number of donors and allografts processed by the different HVBs varied extensively both between and within jurisdictions. Donor numbers ranged from 4 to over 3700, with 8 to 8500 allografts processed annually. Many HVBs in Europe, North America, Australasia, and South Africa have less than 50 donors annually. In contrast, there are two, very large HVBs in North America, which processe allografts from more than 1000 donors each year (Table 10). 

On average, 70% of the processed allografts (range = 39%–90%; mean = 69%) met the HVBs' release criteria. Twenty-one HVBs (21 of 23 banks = 91%) reported positive microbiology results as a primary reason for allografts failing to meet release criteria. Ten (10 of 23 banks = 43%) listed abnormal morphology of tissues, which is defined as presence of calcification, excessive atheroma (particularly in aortic valves), excessive fenestrations, fibrosis, dilatation, and sinus aneurysm, as a reason for product failure. Positive serology results of donor, valve incompetency during testing, technical issues, quality, and donor related findings (e.g., medical contraindication) were also cited as reasons for product failure. One large North American HVB deferred further processing of donor tissues based on low clinical demand of a certain valve type (i.e., aortic valve) and valve sizes (Table 11). 

On average, 85% of allografts meeting release criteria (range = 50%–100%) were implanted. Uncontrollable factors such as low demand for aortic valves, nonvalve allografts, and certain valve sizes were cited as the primary reason for released products not being implanted by 17 HVBs (17 of 23 banks = 74%). The majority of these valves were discarded due to reaching maximum storage duration. Clinical decision during surgery was also cited as a reason why valves were not implanted. Other factors, which could be monitored and potentially improved upon, include perioperative incidents, such as incorrect valve size delivered to implant hospitals, damage during thawing, and intraoperative contamination of allograft. Four HVBs cited these factors as reasons that prevented implantation of released allografts (4 of 23 banks = 17%) (Table 11).

## 4. Discussion

Based on the information published in 2004, there are 85 HVBs worldwide, with the majority located in Europe, Canada, USA, and Australia. There are only three HVBs in Asia [5]. From the responses collated from 24 of these HVBs located in various jurisdictions, it is obvious that global harmonisation of heart valve manufacturing procedures has not been achieved. This is not surprising, as each country has different microbial, environmental, regulatory, and logistical challenges to overcome. Despite so, the more routine processing aspects appeared to be relatively standardised. These included (1) the use of sterile culture media for all processing steps, with the majority of banks using either M199 or RPMI-1640, (2) the use of DMSO as a cryoprotectant, with 72% of the banks utilising this reagent at a concentration of 9%–11%, (3) bioburden reduction by antibiotic disinfection, (4) controlled rate freezing for cryopreservation of allografts, and (5) storage of allografts at ultralow temperatures of below −135°C or in liquid nitrogen vapour phase.

However, in terms of procedural details, numerous differences were also identified. They included (1) type of sterile culture media used for processing, (2) antibiotics combination, (3) temperature and duration used for bioburden reduction, (4) concentration of DMSO used for cryopreservation, and (5) storage duration for released homografts. These differences in processes, which in some cases had been patented by the individual HVBs, had been validated.

Antibiotic cocktails used for bioburden reduction were diverse in terms of the number, combination, and concentrations of antibiotics used. Vancomycin and gentamicin were the most commonly used antibiotics, with approximately 80% of HVBs using one or both of these antibiotics. It is noteworthy that some HVBs were found to prefer first-generation antibiotics, such as penicillin and streptomycin, whereas others chose to use newer, broader spectrum antimicrobials, such as vancomycin and gentamicin. The decision to retain the antibiotics cocktail is probably because the effectiveness of these antibiotics had been validated by the HVBs and no protocol change was warranted. However, as continuous improvement in tissue manufacturing is encouraged, it is important that HVBs routinely review their antibiotic regimens, concentrations, and incubation conditions, to ensure optimal product quality, function, and safety. 

The incubation temperature used for bioburden reduction also varied. Interestingly, there appeared to be a regional preference for different tissue incubation temperatures. This trend had previously been reported by Parker. In his study, he found that most tissue banks in Europe and North America treated allografts with antibiotics at 4°C, whereas banks in Australia conducted antibiotics decontamination step at 37°C [6]. Our survey confirmed these findings, with most banks in Europe and North America incubating recovered donor tissues at 1°C–10°C for 18–24 hours and the majority of banks in Australasia incubating at 35°C–37°C for 6–12 hours. One Australasian HVB had validated both temperatures (4°C and 37°C) for varying durations (18–24 hours and 6–8 hours, resp.) to ensure that processing could be completed within the required time frame.

Differences in duration and temperature of bioburden reduction step, as well as the combination and concentration of antibiotics used by individual HVBs, can be attributed to a number of factors. Firstly, the fact that each procedure had been validated, in some cases patented, and shown to be effective in removing detectable microbial contamination from the allografts, justifies the retention of both antibiotic cocktail and incubation procedure for each HVB. In addition, differences in endemic microorganisms sensitive to different antibiotics are also valid reasons for maintaining these procedural differences between different HVBs. 

Although different antibiotics function optimally within specific temperature ranges and possess varying degrees of stability, maximal antibiotic activity is generally achieved at a physiological temperature of 37°C, when most micro-organisms are actively replicating [7, 8]. A comprehensive study conducted in 2010 by Germain et al. demonstrated that exposure to antibiotics at 37°C resulted in a rapid decrease in the number of colony-forming units of 12 bacterial strains [8]. In addition, the results of a recent study by Fan et al. suggested that the lower decontamination success rate observed when bioburden reduction step was conducted at 4°C was because the micro-organisms were not actively replicating at this temperature. This was particularly relevant for slow-growing organisms, such as the common skin contaminant, *Propionibacterium acnes *[9]. However, it is recognised that disinfecting tissues at this lower temperature has the advantages of reducing bacterial proliferation, as well as preventing warm ischemia damage to the tissue [9, 10]. To minimise potential ischemic damage, most HVBs that incubated allografts at 37°C used an incubation time of ≤12 hours [7]. 

It is noteworthy that the antifungal drug, amphotericin B, was included in the antibiotic cocktail of some HVBs, when its usage had been discontinued in others [7, 11]. Deleterious effects of amphotericin B on human tissue viability and cell damage were reported by various studies [12, 13]. However, the necessity to preserve cell viability for optimal graft function remained controversial. There is clinical evidence that viable valves at the time of cryopreservation had a lower level of structural deterioration than that of nonviable valves. Such valves were more likely to retain thicker leaflets and fewer perforations, as compared with nonviable valves [14]. Conversely, studies have reported the loss of cell viability after cryopreservation and thawing, suggesting that viable cells might not contribute to valve function and durability *in situ *[15]. It has even been suggested that the presence of viable cells in the graft may actually increase immunogenicity* in situ* [16, 17]. Nonetheless, the presence of viable cells precryopreservation is recognised as a useful indicator of optimal tissue preservation [14]. 

Despite the extensive variation in antibiotic regimens, reported decontamination success rates remain comparable at between 60% and 70% for different HVBs [9]. This confirmed the results of our survey where the mean proportion of allografts meeting clinical release criteria was found to be 69%. However, when comparing the proportion of valves meeting release criteria achieved by individual HVBs, the results were actually quite disparate (range = 39%–90%). This finding appeared to reflect the stage at which tissue exclusion criteria were applied. Some HVBs might have processed all donors' tissues prior to application of tissue exclusion criteria, while others ensured that the criteria were met prior to processing. One large North American HVB chose not to process valves based on low clinical demand for the valve type (i.e., aortic valve) and/or size, which reduced the proportion of their products released for implant to only 39%.

Storage duration for released valves ranged from 2 to 15 years, with the majority of HVBs (~70%) storing the allografts for 5 years. Some groups advocated that allografts should be stored for ≤5 years. However, this limit was based on convention rather than a validated study. Mirabet et al. concluded that any significant loss of cell viability in tissues stored in liquid nitrogen for ≤13 years was due to freezing and thawing protocols rather than the storage duration [18].

Almost all HVBs cited “positive microbiological culture of graft” as one of the primary reasons for allografts failing to meet release criteria. Several factors, such as the recovery site environment, sequence of tissue recovery, aseptic recovery and processing techniques, and suboptimal effectiveness of antibiotics used for decontamination, could contribute to this result. Fan et al. reported a significantly higher initial contamination rate of arterial tissues compared to heart tissues, which was ascribed to the less sterile state of the abdominal compartment where the arteries were recovered, especially if trauma was involved [9]. The Prince Charles Hospital also reported high contamination rates when they initiated valve retrieval from multiorgan donors. A review of procedures found contamination to be more common if previous retrieval teams had perforated the bowel or the heart had been removed with instruments used to retrieve other tissues. A higher incidence of contamination has also been reported for valves recovered in an open mortuary area due to the reduced air quality of the mortuary environment [7]. Given the numerous sources for potential contamination, effective aseptic practices at all stages of tissue recovery and processing are essential to minimise the risk of tissue contamination and loss of products.

Over the last thirty years, there has been a significant change in clinical demand for allografts from aortic valves to pulmonary valves. The reasons for this change include an increase in paediatric cardiac surgery, the adoption of the Ross procedure, and improved durability of aortic porcine valves [19, 20]. Various reports also associated the use of aortic valves with more rapid failure than pulmonary valves [21, 22]. The reduced clinical demand for aortic valves, along with low demand for certain valve sizes, were cited as the primary reasons why released valves were not implanted by the majority of HVBs. Together, these valves represented the majority of grafts discarded due to maximum storage duration attained. Other graft types, such as patches, face a similar challenge due to the ready availability of cost-effective animal-sourced patches. 

There are several limitations to this study. Firstly, there were only 24 HVBs, which participated in this survey. This means that the processing practices and outcomes of many other HVBs remain unknown. Secondly, although all HVBs performed formal validation on major processes, the test methodologies used were different. Thirdly, there is a lack of patient-related parameters, which are more accurate measurements to the quality of final products and, ultimately, the success of transplanted allografts. Ideally, parameters such as early leaflet failure, iatrogenic infection, and other perioperative adverse events of patients, due to processing variables and thawing protocols, should be reviewed. While beyond the scope of this paper, a separate study should be performed to address these issues and potentially identify practical solutions.

Although the findings of this survey suggested that procedures currently in use by the individual HVBs were validated, in consideration of the wide variation in percentage of allografts fulfilling release requirements (range = 39%–90%), some purportedly validated protocols may not be performing as optimally as others. This seems to present an opportunity for improvement in the heart valve banking industry. As underperforming protocols will incur substantial losses to HVBs, all efforts to improve HVBs' processing activities and recipient outcomes should be encouraged. Although “global harmonisation” is a worthy goal, it is doubtful if this can be achieved in the heart valve banking industry for a number of reasons, which include patented processes, organisational restrictions, and environmental differences. However, regular reviews of important outcomes as cited in this paper and monitoring of adverse patient events should encourage comparison and improvements in the HVBs' processes.

## Figures and Tables

**Table 1:** Summary of heart valve processing protocols used by HVBs in Europe.

Bank number	Processing media	Antibiotic regimen	Incubation protocol	Cryopreservation method	Storage condition and duration
E1	Until 2004: M199 2004 onwards: RPMI-1640	Vancomycin: 500 ug/mL Gentamycin: 50 ug/mL Piperacillin: 500 ug/mL Nystatin: 2500 U/mL	Room temperature, 24 hours, in the dark	Controlled rate freezing	Liquid nitrogen vapour phase: 5 years

E2	RPMI-1640 with glutamine Addition of 20% human albumin and DMSO for cryopreservation and storage	Cefoxitin: 240 ug/mL Lincomycin: 120 ug/mL Colimycin: 100 ug/mL Vancomycin: 50 ug/mL	4°C, 24 hours	Controlled rate freezing from +10°C to −110°C	Liquid nitrogen vapour phase: 5 years

E3	Transport solution: TRIS buffered isotonic saline Antibiotics media: M199 Cryopreservation media: HBSS with 25 mM HEPES + 20% DMSO	Gentamicin: 4000 ug/mL Imipenem: 200 ug/mL Nystatin: 2500 U/mL Polymyxin B: 200 ug/mL Vancomycin: 50 ug/mL	2–8°C, 18–24 hours	Controlled rate freezing (at −1°C/min)	−135°C: 10 years

E4	Cryopreservation media: HBSS with 25 mM HEPES + 10% DMSO	Vancomycin: 50 ug/mL Gentamicin: 4000 ug/mL Ciprofloxacin: 200 ug/mL Amphotericin B: 50 ug/mL	Room temperature (21°C), 24 hours	Controlled rate freezing	Liquid nitrogen vapour phase: 10 years

E5	Antibiotics media: RPMI-1640 Cryopreservation media: RPMI-1640 + 10% DMSO	Metronidazol: 50 ug/mL Vancomycin: 50 ug/mL Amikacin: 50 ug/mL Amphotericin B: 5 ug/mL	4°C, 24 hours	Controlled rate freezing	Liquid nitrogen vapour phase: 15 years

E6	Cryopreservation media: 70% TC-199 + 10% DMSO + 20% human albumin	Vancomycin: 50 ug/mL Tobramycin: 50 ug/mL Cotrimoxazole: 50 ug/mL	4°C, 6–24 hours	Controlled rate freezing (at −1°C/min)	Liquid nitrogen vapour phase: 10 years

E7	Cryopreservation media: M199 in the past, use sodium chloride solution + 10% DMSO at present	Fluconazole: 200 mg Cefotaxime: 1 g	4°C, 24 hours	Liquid nitrogen vapour phase	Liquid nitrogen vapour phase: 2 years

E8	Antibiotic media: 0.9% sodium chloride Cryopreservation media: RPMI-1640 + 10% cryoprotectant	Metronidazole: 20 ug/mL Gentamicin: 20 ug/mL Flucloxacillin: 20 ug/mL	4°C, at a minimum of 12 hours	Controlled rate freezing (down to −180°C)	Liquid nitrogen vapour phase: 5 years

E9	M199 with Hanks' salts, L-glutamine, 25 mM HEPES	Lincomycin, polymyxin B sulphate, vancomycin	4°C, 48 hours	No response provided	Liquid nitrogen vapour phase: 5 years

E10	M199 Antibiotics media: 200 mL of Earles salt in M199	Amphotericin B: 250 ug/mL Fungoral: 100 ug/mL Colistin: 200 ug/mL Vancocin: 500 ug/mL Garamycin: 530 ug/mL	4°C–8°C, 24 hours	Controlled rate freezing	Liquid nitrogen vapour phase: 10 years

E11	Transport solution: Ringer's lactate or cardioplegia solution Antibiotic media: M199 Cryopreservation media: M199 + Antibiotics + 12.5% DMSO	Cefuroxime: 250 ug/mL Gentamicin: 80 ug/mL Ciprofloxacin: 200 ug/mL Vancomycin: 500 ug/mL Colistin: 1000 IU/mL Amphotericin B: 20 ug/mL	37°C, 18–24 hours	Controlled rate freezing (from +4°C to −60°C at 1°C per min; then transfer to ultralow temperature freezer at −140°C)	Ultralow temperature (−140°C): 5 years or −80°C: for up to 6 months

**Table 2:** Summary of heart valve processing protocols used by HVBs in North America.

Bank number	Processing media	Antibiotic regimen	Incubation protocol	Cryopreservation method	Storage condition and duration
N1	Heart recovery, transport, and dissection solution: Ringer's lactate Antibiotics media: DMEM with Hepes Cryopreservation media: DMEM + 7.5% DMSO	Vancomycin: 50 ug/mL Gentamicin: 80 ug/mL Cefoxitin: 240 ug/mL	Until the 28th of June 2010: 1°C–10°C, 22–26 hours Present: 33°C–38°C, 18–26 hours	Controlled rate freezing	No response provided

N2	Antibiotics media: RPMI-1640 Cryopreservation media: X-VIVO-10 + DMSO	Cefoxitin, colymycin-M, vancomycin, lincomycin	4°C, 24 hours	Controlled rate freezing	Liquid nitrogen vapour phase: 5 years

N3	Transport and storage solution: Hanks solution Dissection solution: saline Cryopreservation media: 10% DMSO	Gentamicin: 80 mg/mL Cefazolin or Kefzol: 1 mg/mL	4°C, 24 hours	Controlled rate freezing	Ultralow temperature (−140°C): 5 years

N4	Transport and processing solution: Ringer's lactate Cryopreservation media: saline + RPMI-1640 + RPMI 1640 with 10% FBS + DMSO	Cefoxitin: 240 ug/mL Polymyxin B: 100 mg/mL Vancomycin: 50 ug/mL Lincomycin: 120 ug/mL (soon to use gentamicin)	1°C–10°C, 22–26 hours	Controlled rate freezing	Liquid nitrogen vapour phase: 5 years

N5	DMEM Cryopreservation media: cardiac tissue—DMEM + DMSO + FBS vascular tissue—DMEM + DMSO + chondroitin + FBS	Two-stage process: 1st cocktail to achieve primary decontamination, 2nd cocktail to support tissue	Warm solutions, >24 hours	Controlled rate freezing	Liquid nitrogen vapour phase: 5 years

N6	RPMI-1640	Vancomycin: 50 ug/mL Colymycin M: 75 mg/mL Cefoxitin: 100 mg/mL Lincomycin: 300 mg/mL	4°C, 24 ± 2 hours	Controlled rate freezing	Liquid nitrogen vapour phase: 5 years

**Table 3:** Summary of heart valve processing protocols used by HVBs in Australasia and South Africa.

Bank number	Processing media	Antibiotic regimen	Incubation protocol	Cryopreservation method	Storage condition and duration
A1	Heart collection, transport, dissection solution: Hartmann solution Antibiotic media: M199 Cryopreservation media: M199 + 10% DMSO	Amoxicillin: 20 ug/mL Gentamicin: 20 ug/mL	37°C, 6–8 hours or 4°C, 18–24 hours	Controlled rate freezing	Liquid nitrogen vapour phase: 5 years

A2	Heart collection, transport, dissection solution: saline or M199 Antibiotic media: M199 Cryopreservation media: M199 + 10% DMSO	Penicillin: 50 IU/mL Streptomycin: 50 ug/mL	35°C, 6–8 hours	Controlled rate freezing (back up: manual controlled rate freezing)	Liquid nitrogen vapour phase: 5 years

A3	Heart collection, transport, dissection solution: DCD/multiorgan donors/live donors—saline Cadaveric—saline or M199 with antibiotics Cryopreservation media: M199 + 10% DMSO	Penicillin: 50 IU/mL Streptomycin: 50 ug/mL	37°C, 6–12 hours	Controlled rate freezing	Liquid nitrogen vapour phase: 10 years

A4	Heart collection, transport, dissection solution: Hartmann solution Antibiotic media: M199 Cryopreservation media: M199 + 10% DMSO	Benzylpenicillin: 30 ug/mL Gentamicin: 18 ug/mL	37°C, 6 hours	Controlled rate freezing	Liquid nitrogen vapour phase: 5 years

A5	Heart collection, transport, dissection, rinse solution: HBSS (preferred) or saline Antibiotic media: HBSS Cryopreservation media: Until Jan 2010—M199 + 10% DMSO After Jan 2010—HBSS + 10% DMSO	Cefoxitin: 240 ug/mL Lincomycin: 120 ug/mL Polymyxin B: 100 ug/mL Vancomycin: 50 ug/mL Amphotericin B: 25 ug/mL	First soak: 4°C, 24 hours; 2nd soak: 4°C, 24 hours; transfer to HBSS at 4°C until frozen	Freezing in liquid nitrogen vapour—no controlled rate equipment used	Liquid nitrogen vapour phase: 5 years

A6	Transport, dissection, incubation solution: M199 Cryopreservation media: M199 + 100% DMSO diluted to 10%	Vancomycin: 50 ug/mL Amikacin: 100 ug/mL	4°C, 24 hours	Controlled rate freezing	Liquid nitrogen vapour phase: 5 years

A7	M199 11% DMSO	Mefoxin: 50 ug/mL Piperacillin: 50 ug/mL Amikacin: 25 ug/mL Amphotericin B: 2.5 ug/mL	4°C, 12–18 hours	Controlled rate freezing	Liquid nitrogen vapour phase: 5–8 years

**Table 4:** Types and concentrations of antibiotics used for decontamination of allografts.

Antibiotics/antifungals	Concentration/mL	Number of HVBs
Vancomycin/vancocin	50–500 ug	15
Gentamicin/garamycin	18–4000 ug	10
Cefoxitin/cefotaxime/cefuroxime	240–1000 ug	6
Amphotericin B	2.5–50 ug	5
Lincomycin	120 ug–300 mg	3
Penicillin/benzylpenicillin	30–50 IU	3
Amikacin	25–100 ug	3
Polymyxin B	200–1000 ug	3
Piperacillin	50–500 ug	2
Colistin	200 ug/1000 IU	2
Streptomycin	50 ug	2
Tobramycin	50 ug	1
Cotrimoxazole	50 ug	1
Fluconazole	500 mg	1
Flucloxacillin	200 ug	1
Fungizone	250 ug	1
Fungoral	100 ug	1
Imipenem	200 ug	1

**Table 5:** Types of incubation media used for decontamination of allografts.

Incubation media	Number of HVBs	% of HVBs
Medium 199 (TC-199/M199)	11	46%
Roswell Park Memorial Institute Media 1640 (RPMI-1640)	5	21%
0.9% sodium chloride (normal saline)	3	12.5%
Dulbecco's Modified Eagle's Medium (DMEM)	2	8%
Hanks Balanced Salt Solution (HBSS)	2	8%
Ringers lactate	1	4.5%

**Table 6:** Incubation temperatures and durations used for decontamination of allografts.

Incubation temperature (in °C)	Incubation time (in hours)	Number of HVBs	Number of HVBs	% of HVBs
1–10 (cold temperature, including at 4°C)	6–24	1	16	67%
	12–18	2		
	18–26	12*		
	48	1		
21 (ambient temperature)	18–26	2	2	8.3%
33–38 (physiological temperature)	6–12	4*	7	29%
	18–26	3		

*Refers to a HVB which decontaminates allografts at either 37°C for 6–8 hours or 4°C for 18–24 hours.

**Table 7:** Summary of heart valve outcomes for HVBs in Europe.

Bank	Number of donors/number of grafts processed annually (Average)	Proportion of products meeting release criteria	Reasons for product failure	Proportion of released products implanted	Reasons released products are not implanted
E1	45 donors 75 valves	~79%	(1) Abnormal morphology of graft (2) Positive microbiological results of graft (3) Positive serology results of donor	99.5%	Decision during surgery

E2	25 donors 40 valves	~70%	(1) Failure in valve competency (2) Positive microbiological results of graft (3) Technical issues	95%	Decision during surgery

E3	97 grafts	~50%	Positive microbiological results	95%	(1) Size demands for valves (2) Low demand for type of valve

E4	72 donors 80 valves 130 patches	90%	(1) Failure in valve competency (primarily aortic valves) (2) Positive microbiological results of graft	98%	Decision during surgery, cancellation of surgery

E5	4 donors 8 valves	~69%	(1) Positive microbiological results of graft (2) Positive serology results of donor	100%	NA

E6	35 donors 30 valves	~70%	Positive microbiological results of graft	95%	Low demand for aortic valves

E7	20–30 donors 50 valves	~90%	(1) Positive microbiological results of graft (2) Technical issues	60%	Size demand for valves—a congenital cardiac centre only

E8	40–50 donors 80 valves	~80%	Positive microbiological results of graft	95%	(1) Size demand for valves (2) Beyond maximum storage duration of patch graft

E9	300 donors 400 valves	~50%	(1) Abnormal morphology of graft (2) Positive microbiological results of graft (3) Technical issues	85% (100% for pulmonary valves and arterial allografts)	(1) Aortic valve size (2) Low demand for aortic valves (3) Decision during surgery (4) Damage of graft during thawing

E10	100 donors 250 grafts	~68%	Abnormal morphology of graft	65%	Size demand for valves

E11	217 donors 380 valves	~78%	(1) Abnormal morphology of graft (2) Positive microbiological results of graft	84%	(1) Size demand for valves (2) Low demand for aortic valves

**Table 8:** Summary of heart valve outcomes for HVBs in North America.

Bank	Number of donors/no. of grafts processed annually (Average)	Proportion of products meeting release criteria	Reasons for product failure	Proportion of released products implanted	Reasons released products not implanted
N2	55 donors 70 valves	~70%	(1) Abnormal morphology of graft (2) Positive serology results of donor (3) Positive microbiological results of graft (4) Donor related	100% for pulmonary valves 20% for aortic valves	Low demand for aortic valves

N3	42 donors 68 grafts	~78%	Positive microbiological results of graft	32% of grafts were confirmed to be implanted, 16.5% of grafts were exported	Low demand for aortic valves

N4	2400 grafts	~60%	(1) Positive microbiological results of graft (2) Quality/donor related	80%	(1) Size demand for valves (2) Nonvalve grafts are not in demand

N5	3700 donors 8500 grafts (including valves, patches, descending thoracic aorta and pericardium patches)	~39%	(1) Deferred due to size/type of graft (2) Graft attribute/abnormal morphology of graft (3) Positive microbiological results of graft	99%	Beyond maximum storage duration

N6	34 donors 57 grafts (valves, conduits, and pulmonary hemiarteries)	No response provided		53%	(1) Intraoperative contamination of graft (2) Incorrect size of graft (3) Beyond maximum storage duration

**Table 9:** Summary table of heart valve outcomes for HVBs in Australasia and South Africa.

Bank	Number of donors/number of grafts processed annually (average)	Proportion of products meeting release criteria	Reasons for product failure	Proportion of released products implanted	Reasons released products are not implanted
A1	12 donors 16 valves	~75%	(1) Positive microbiological results of graft (2) Abnormal morphology of graft	77%	(1) Size demand for aortic valves (2) Low demand for aortic valves

A2	27 donors 77 grafts	~50%	(1) Positive microbiological results of graft (2) Donor related (medical contradiction)	90–100%	Size demand for aortic valves

A3	80 donors 232 grafts (118 valves and 114 patches)	~70%	(1) Positive microbiological results of graft (2) Positive serology results of donor	100% for pulmonary valves; majority for aortic valves; 100% for patches	(1) Size demand for aortic valves (2) Beyond maximum storage duration

A4	20 donors 36 valves	~83%	(1) Positive microbiological results of graft (2) Abnormal morphology	74%	Low demand for aortic valves

A5	24 donors 34 valves	90%	Positive microbiological results of graft	~100%	(1) Quality of graft (2) Beyond maximum storage duration

A6	29 donors 49 valves	~71%	(1) Positive microbiological results of graft (2) Abnormal morphology (3) Failure in valve competency	91%	Nonvalve grafts are not in demand

A7	75 donors 98 valves	~61%	(1) Positive serology results of donor (2) Positive microbiological results of graft (3) Abnormal morphology of graft	~80%	Size demand for valves

**Table 10:** Number of donors processed by HVBs, according to geographical region.

Number of donors	Europe	North America	Australasia and South Africa	Total
<50	7	2	5	14
51–100	2	1	2	5
101–1000	2	0	0	2
>1000	0	2	0	2

**Table 11:** Reasons for product failure and why released products were not implanted.

Reasons for product failure	Number of HVBs	Reasons why released products were not implanted	Number of HVBs
During evaluation of tissue and processing		Uncontrollable factors	
Abnormal morphology	10	Size demands for valves	11
Failure in valve competency	3	Aortic valve/nonvalve products not in demand	10
Technical issues	3	Beyond maximum storage duration	5
Deferred due to size/type of graft	1	Decision during surgery	4

After tissue processing		Factors that can be improved on	
Positive microbiological results of graft	21	Damage of graft during thawing	1
Positive serology results of donor	5	Intraoperative contamination of graft	1
Quality/donor related	3	Incorrect size of graft	1
		Quality of graft	1
